# P-1523. Evaluation of Standardized Treatment Regimens for Patients with Documented Melioidosis Septicemia at Tertiary Care Hospital in Thailand

**DOI:** 10.1093/ofid/ofae631.1692

**Published:** 2025-01-29

**Authors:** Korawan Pudpong, Tipanong Gatechan

**Affiliations:** Sunprasitthiprasong Hospital, Meung, Ubon Ratchathani, Thailand; Sunprasitthiprasong Hospital, Meung, Ubon Ratchathani, Thailand

## Abstract

**Background:**

*Burkholderia pseudomallei* (*B. pseudomallei*), an environmental Gram-negative bacterium, is the causative agent of melioidosis in humans. Therefore, its biothreat potential, the U.S. Centers for Disease Control and Prevention designates it as a tier 1 select agent.

Melioidosis is predominantly a disease in subtropical and tropical regions. It is endemic in northern Australia and parts of Southeast Asia and the Indian subcontinent. Mostly, melioidosis (85%) results in acute infections from recent bacterial exposure. The majority of patients with acute melioidosis present with sepsis with or without pneumonia, or localized abscesses, regardless of the route of infection.

Currently, therapy consists of an intensive phase of a minimum of 10 days with intravenous ceftazidime or a carbapenem (meropenem or imipenem), with or without trimethoprim-sulfamethoxazole to prevent mortality followed by an eradication phase with oral antibiotics to prevent relapse.

In Thailand, still found the mortality rate was 40%.We have uncleared study clinical outcomes comparable to ceftazidime and carbapenems treatment in documented melioidosis septicemia.

This is the first study to evaluate the efficacy of ceftazidime compared to carbapenems in patients with documented melioidosis septicemia whose blood cultures confirmed *B. pseudomallei* and susceptible to both ceftazidime and carbapenems in Thailand.

Baseline characteristic (N=264)

**Figure:**
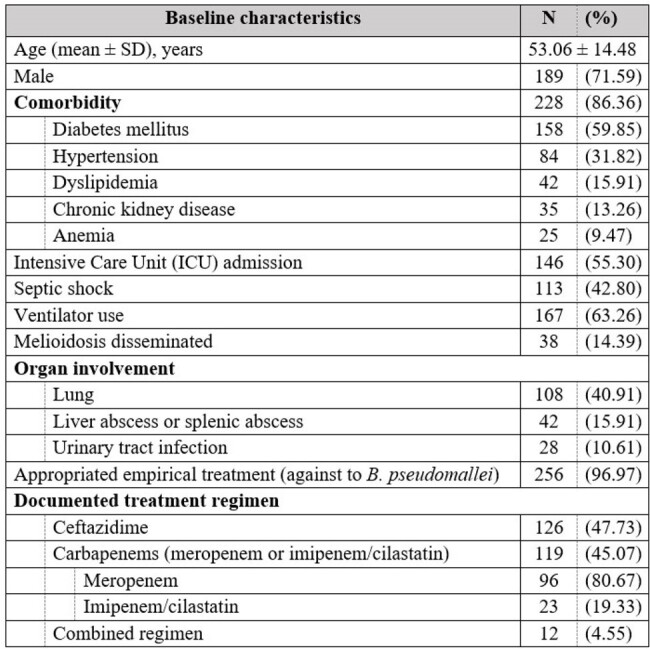

**Methods:**

A retrospective study was conducted at Sunprasitthiprasong hospital, Thailand from 2021-2023. Adult patients who were admitted had documented positive *B. pseudomallei* septicemia and received ceftazidime or carbapenem at least one dose. The exclusion criteria were incomplete chart summary. Significant level p-value≤0.05 (STATA version 16)

ceftazidime versus carbapenems in non-septic shock patients

**Figure:**
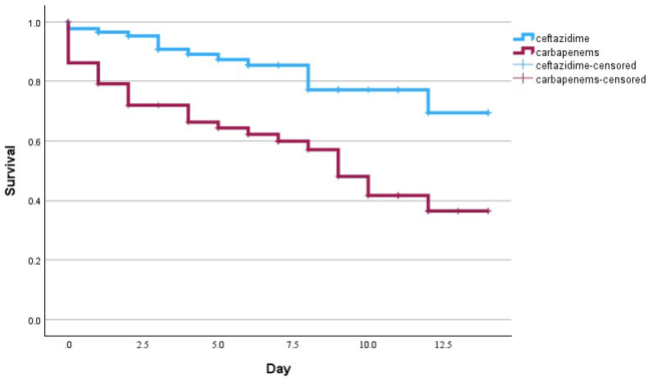

p-value < 0.001

**Results:**

264 patients were included and reported *B. pseudomallei* septicemia susceptibility to ceftazidime and meropenem were 264 (100%) pathogens and imipenem/cilastatin 262 (99.24%) pathogens.

ceftazidime versus carbapenems in septic shock patients

**Figure:**
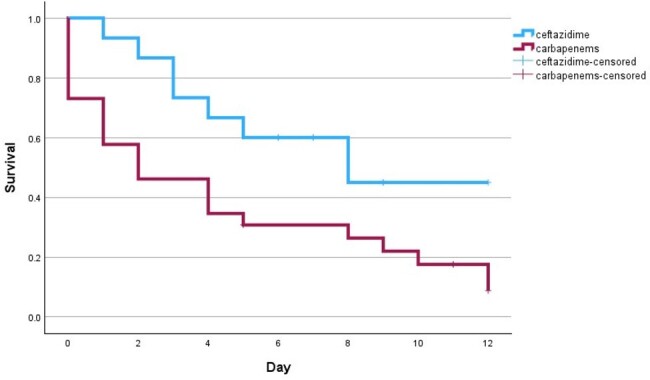

p-value =0.034

**Conclusion:**

Ceftazidime compared to carbapenems in the intensive phase for documented melioidosis septicemia trends higher survival rate in 14 days after confirming pathogens in non-severe patients. Furthermore, randomized controlled trials (RCTs) will prove clinical outcomes in documented melioidosis septicemia.

Factors associated with mortality

**Figure:**
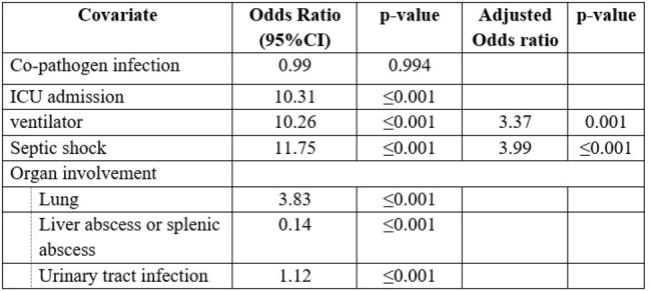

**Disclosures:**

**All Authors**: No reported disclosures

